# Numerical Investigation of Perovskite/Silicon Heterojunction Tandem Solar Cell with a Dual-Functional Layer of MoO_X_

**DOI:** 10.3390/ma18071438

**Published:** 2025-03-24

**Authors:** Tian-Yu Lu, Jin Wang, Xiao-Dong Feng

**Affiliations:** College of Materials Science and Engineering, Nanjing Tech University, Nanjing 211816, China; 202261103030@njtech.edu.cn (T.-Y.L.); 202361103043@njtech.edu.cn (J.W.)

**Keywords:** tandem device, MoO_X_, recombination layer, simulation, Silvaco TCAD

## Abstract

This study proposed a novel perovskite/silicon heterojunction (SHJ) tandem device structure without an interlayer, represented as ITO/NiO/perovskite/SnO_2_/MoO_X_/i-a-Si:H/n-c-Si/i-a-Si:H/n-a-Si:H/Ag, which was investigated by Silvaco TCAD software. The recombination layer in this structure comprises the carrier transport layers of SnO_2_ and MoO_X_, where MoO_X_ serves dual functions, acting as the emitter for the SHJ bottom cell and as part of the recombination layer in the tandem cell. First, the effects of different recombination layers are analyzed, and the SnO_2_/MoO_X_ layer demonstrates the best performance. Then, we systematically investigated the impact of the carrier concentration, interface defect density, thicknesses of the SnO_2_/MoO_X_ layer, different hole transport layers (HTLs) for the top cell, absorption layer thicknesses, and perovskite defect density on device performance. The optimal carrier concentration in the recombination layer should exceed 5 × 10^19^ cm^−3^, the interface defect density should be below 1 × 10^16^ cm^−2^, and the thicknesses of SnO_2_/MoO_X_ should be kept at 20 nm/20 nm. CuSCN has been found to be the optimal HTL for the top cell. When the silicon absorption layer is 200 μm, the perovskite layer thickness is 470 nm, and the defect density of the perovskite layer is 10^11^ cm^−3^, the planar structure can achieve the best performance of 32.56%. Finally, we studied the effect of surface texturing on the SHJ bottom cell, achieving a power conversion efficiency of 35.31% for the tandem cell. Our simulation results suggest that the simplified perovskite/SHJ tandem solar cell with a dual-functional MoO_X_ layer has the potential to provide a viable pathway for developing high-efficiency tandem devices.

## 1. Introduction

Crystalline silicon cells have dominated the solar market with a maximum efficiency of 27.3% [1], which is near their theoretical limit [2]. To surpass this limit, tandem cells have emerged as a key solution. This approach utilizes two or more single-junction cells stacked together to efficiently absorb the solar spectrum. Currently, the efficiency of single-junction perovskite cells has rapidly advanced, from 3.8% [3] in 2009 to 26.7% [4]. They exhibit superior performance, tunable energy bands, simple preparation, and low cost [5]. Naturally, the combination of perovskite and crystalline silicon cells in tandem devices has been an important research area. In fact, perovskite is widely used as the top cell in tandem solar cells, paired with bottom cells such as silicon [6], CIGS [7], organic device [8], and other perovskite cells [9] to form various tandem configurations. Among these tandem devices, perovskite/silicon cells have shown rapid progress in efficiency due to significant advances in material selection and structural optimization of top perovskite cells [10,11,12]. Their efficiency has been raised from an initial 13.7% [13] to 34.6% [14]. Except for the top cell, the design of the recombination layer is also critical for tandem devices, as it must facilitate both optical transmission and electrical connection [15].

Currently, there are three design strategies for the recombination layer for perovskite/silicon tandem devices. The most commonly used material in the recombination layer of tandem cells is transparent conductive oxide (TCO), such as indium tin oxide [16] (ITO), Indium Zinc Oxide [17] (IZO), and aluminum-doped zinc oxide [18] (AZO). Nevertheless, TCOs are susceptible to optical loss when it comes to ensuring high conductivity [19]. The second strategy is to employ silicon-based tunneling junctions, which are heavily doped in nano-crystalline silicon (nc-Si:H) [20,21]. These junctions offer high conductivity, excellent carrier selectivity, and reduced optical losses compared to TCOs. However, the preparation process of tunnel junctions is relatively complex and may affect the low-temperature process of SHJ bottom cells [22]. The third strategy is the interlayer-free design, which enables carrier extraction and recombination by completely removing the conventional recombination layer, allowing direct connection between the top and bottom cell’s the electron transport layer (ETL) or hole transport layer (HTL). This method simplifies the cell structure, reduces preparation costs, and avoids the optical loss of the conventional recombination layer.

The perovskite/silicon tandem solar cells without an interlayer have been reported [23,24,25,26], where the bottom cell is typically a homojunction, such as a Passivated Emitter and Rear Cell (PERC). SnO_2_ serves as both the ETL for the perovskite top cell and part of the recombination layer, forming an ohmic contact with the P⁺-Si emitter of the PERC bottom cell [27]. In this configuration, SnO_2_ functions as an electron transport, while the P⁺-Si layer is responsible for hole transport, enabling electron–hole recombination at the interface. Similarly, TiO_2_ has been employed as an ETL and recombination junction in perovskite/silicon tandem solar cells, where its impurity states assist recombination at the interface [28]. In these interlayer-free tandem devices, the silicon bottom cell is always a homojunction, where minority carriers tend to recombine at the interface, reducing the open-circuit voltage (V_OC_) [29]. In contrast, a silicon heterojunction (SHJ) cell can prevent minority carriers from recombining at the interface, thereby preserving the V_OC_ of the silicon cell [30]. Therefore, SHJ cells can be used as better bottom ones which generally offer superior performance in tandem devices. In conventional perovskite/SHJ tandem cells, the interlayer is often a tunneling junction. However, nc-Si:H used in tunneling junctions leads to parasitic absorption due to its small bandgap [31]. Additionally, the amorphous silicon thin-film emitter of the SHJ cell absorbs some sunlight, also contributing to optical loss. Transition metal oxides (TMOs) can replace a-Si:H to address this issue [32]. For n-type SHJ bottom cells, MoO_X_, which has a high work function [33] and wide bandgap [34], can replace the doped p-type amorphous silicon layer as an emitter, reducing parasitic absorption and improving overall cell performance [35,36]. MoO_X_ is also widely used as an HTL in single-junction perovskite cells. Moreover, it plays a role in the recombination layer in tandem cells such as all perovskite and perovskite/organic [8,37]. In these tandem cells, MoO_X_, together with Ag and/or ITO, forms the recombination layer. In perovskite/SHJ tandem cells, the interlayer-free design allows the electron transport layer (SnO_2_) of the top cell to be deposited directly on the MoO_X_ layer, forming the recombination layer and eliminating the optical losses associated with tunnel junctions. In this configuration, holes collected by MoO_X_ and electrons collected by SnO_2_ can be recombined at their interface. Thus, MoO_X_ plays dual roles as both the emitter for the bottom cell and the recombination layer in the tandem cell. This innovative design has not been reported in perovskite/SHJ tandem devices. Here, our research goal is to develop high-performance tandem devices of perovskite/SHJ.

In this work, Silvaco TCAD [38,39,40] software was employed to simulate perovskite/SHJ tandem devices. First, four recombination layers in the perovskite/SHJ tandem structure are studied. Next, the influence of key parameters on cell performance is analyzed, including the carrier concentration and thickness of the SnO_2_/MoO_X_ recombination layer, the defect density at the SnO_2_/MoO_X_ interface, the HTL of the top cell, the thickness of the absorption layers, and the defect density of the perovskite layer. Finally, the impact of surface texturing on device performance is investigated.

## 2. Methods

### 2.1. Simulation Methodology

The simulation is performed using the Silvaco TCAD tool (version 2019), which is based on three fundamental equations, including the Poisson equation, the drift-diffusion equation, and the carrier continuity equation. By solving these equations, the spatial distributions of parameters such as hole and electron concentrations, electric potential, and others are obtained. These results are used to calculate the current density–voltage (J-V) curves. Then, the relevant electrical parameters of the device can be determined, including open-circuit voltage (V_OC_), short-circuit current density (J_SC_), fill factor (FF), and power conversion efficiency (PCE). The J-V curves are simulated under AM 1.5 G solar spectrum at 1000 W/m^2^ light intensity.

### 2.2. Device Structure

**Figure 1:**
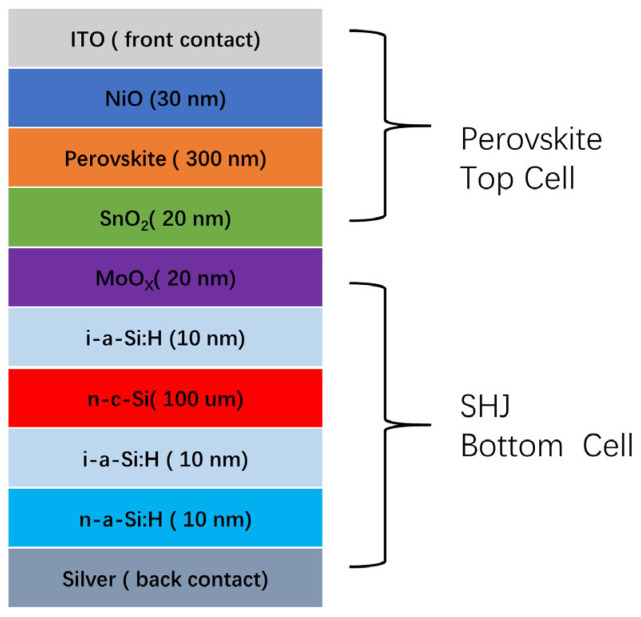
Device structure of the perovskite/SHJ tandem cell.

This tandem solar cell consists of an SHJ bottom cell and a perovskite top cell. As shown in Figure 1, the tandem device structure is as follows: ITO/NiO/perovskite/SnO_2_/MoO_X_/i-a-Si:H/n-c-Si/i-a-Si:H/n-a-Si:H/Ag. The material of FA_0.83_Cs_0.17_PbBr_0.85_I_2.15_, with a bandgap of 1.7 eV [41], is selected as the absorber layer in the top cell. The initial HTL of the top cell is NiO, and the ETL is SnO_2_. MoO_X_ serves dual functions, not only acting as the emitter for the bottom cell but also participating in current recombination with SnO_2_.

Table 1 presents the electrical parameters used in the simulation of the perovskite/SHJ tandem device, sourced from the literature [41,42,43,44] and the Silvaco database. The optical constants (nk values) for NiO, perovskite, SnO_2_, and MoO_X_ were obtained from References [45,46,47,48,49], while those for c-Si, a-Si:H, ITO, and Ag were chosen from the Silvaco database. Table 2 lists the defect parameter settings for the cell. Table 3 outlines the electrical parameters of different HTLs [50], and their optical constants are cited from References [51,52,53].

## 3. Results and Discussion

### 3.1. Effect of Different Recombination Layers

**Figure 2:**
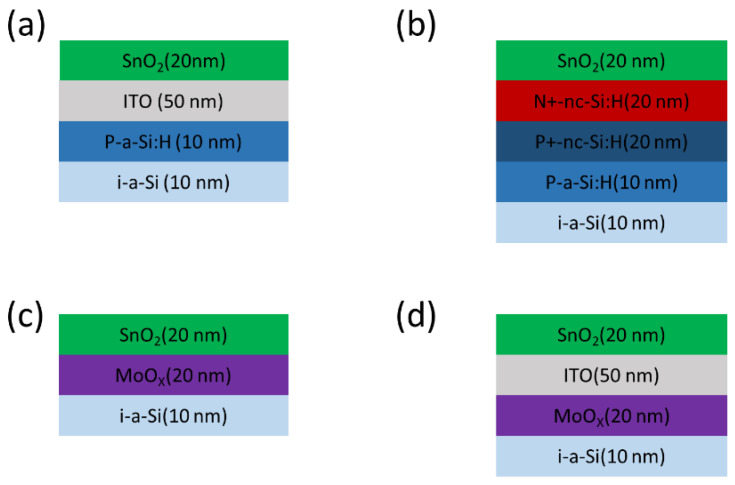
Schematic structure of different recombination layer designs: (**a**) ITO, (**b**) p^+^-nc-Si:H/ n^+^-nc-Si:H, (**c**) SnO_2_/MoO_X_, and (**d**) SnO_2_/ITO/MoO_X_.

Four different structures of the recombination layer were compared, as illustrated in Figure 2. In Figure 2a, the intermediate recombination layer, ITO, is used to connect the top and bottom cells. Figure 2b shows a tunnel junction connecting the top and bottom cell, which includes n^+^-nc-Si:H and p^+^-nc-Si:H. Figure 2c represents the interlayer-free structure, where the ETL (SnO_2_) of the top cell and the HTL (MoO_X_) of the bottom cell are directly connected. Figure 2d shows a structure in which the intermediate layer, ITO, is inserted between MoO_X_ and SnO_2_. The parameters for ITO and nc-Si:H are sourced from the Silvaco database.

**Figure 3:**
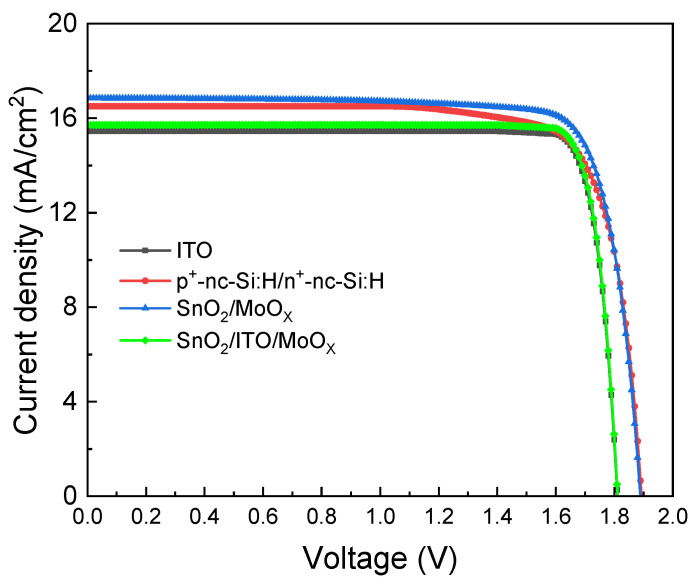
Comparison of J-V curves of tandem cells with different recombination layers.

As shown in Figure 3, the J-V curves demonstrate that the interlayer-free device has the best efficiency. Compared to the ITO and tunnel junction structures, this interlayer-free device reduces parasitic absorption, allowing for more effective light absorption. Regarding V_OC_, as shown in Table 4, the devices with an ITO intermediate layer exhibit a decrease in V_OC_ due to the resistive shunting effect of the ITO layer. Additionally, the devices with the SnO_2_/MoO_X_ recombination layer show a slight reduction in V_OC_ compared to nc-Si:H tunnel junctions. This may be related to insufficient optimization of the recombination layer [23], which leads to the accumulation of un-recombined charges at the SnO_2_/MoO_X_ interface, thereby affecting the overall performance of the device.

### 3.2. Effect of SnO_2_/MoO_X_ Recombination Layer on Device Performance

#### 3.2.1. Effect of the Carrier Concentration of the Recombination Layer

**Figure 4:**
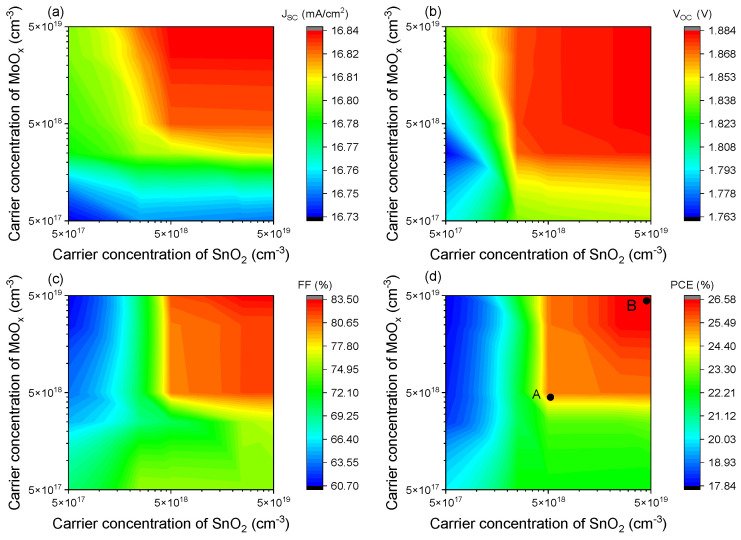
Effect of carrier concentration in the SnO_2_/MoO_X_ recombination layer on device performance of tandem cells: (**a**) J_SC_, (**b**) V_OC_, (**c**) FF, and (**d**) PCE.

The impact of SnO_2_ and MoO_X_ carrier concentrations on tandem device performance has been studied. As shown in Figure 4, an increase in the carrier concentration of SnO_2_ and MoO_X_ from 5 × 10^17^ cm^−3^ to 5 × 10^19^ cm^−3^ results in a monotonic rise in the four parameters: V_OC_, J_SC_, FF, and PCE. Cell performance is poor when the carrier concentration in the recombination layer is less than 5 × 10^18^ cm^−3^. The best efficiency of 26.58% is obtained when the carrier concentration of both the SnO_2_ and MoO_X_ layers reaches 5 × 10^19^ cm^−3^.

**Figure 5:**
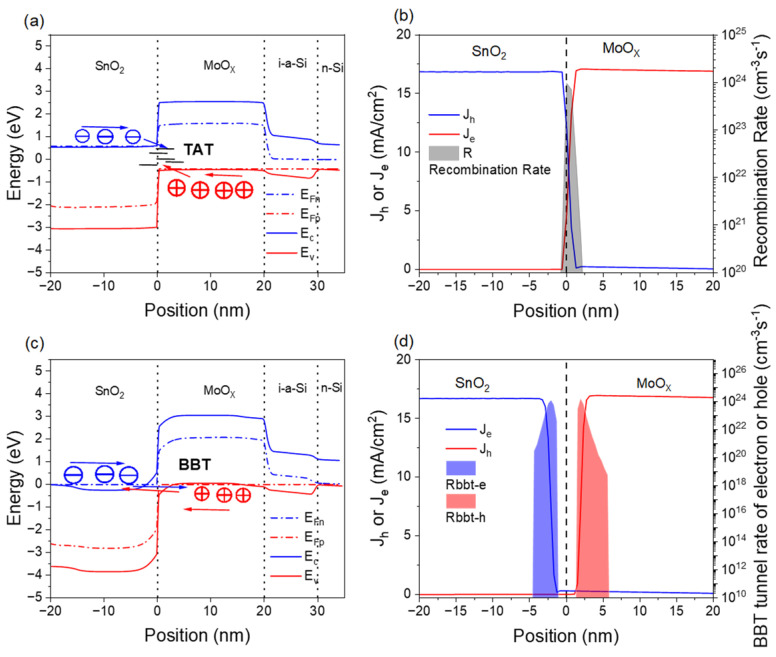
(**a**) Energy band diagram of point A, (**b**) electron–hole current density distribution and recombination rate distribution at point A, (**c**) energy band diagram of point B, and (**d**) electron–hole current density distribution and tunneling rate distribution at point B.

To analyze the mechanism underlying the efficiency improvement with increasing carrier concentration in the recombination layer, points A and B in Figure 4d are selected, corresponding to the carrier concentrations of 5 × 10^18^ cm^−3^ and 5 × 10^19^ cm^−3^, respectively. Figure 5 shows the energy band diagrams and electron (hole) current density distributions for points A and B. The band alignment in the recombination layer determines whether charge transport occurs via band-to-band tunneling (BBT) or a trap-assisted tunneling (TAT) mechanism [54]. In the recombination layer, the carriers in the SnO_2_ layer are primarily electrons, while the carriers in the MoO_X_ layer are holes. The energy band diagram in Figure 5a shows a clear separation between the quasi-Fermi level of electrons in SnO_2_ and that of holes in MoO_X_. There is no overlap between the valence band (E_V_) of MoO_X_ and the conduction band (E_C_) of SnO_2_. From Figure 5b, we observe the electron–hole current density distribution and a high recombination rate at the SnO_2_/MoO_X_ interface. These results suggest that the charge transport in the recombination layer occurs via TAT due to interface defects.

In contrast, Figure 5c shows an energy overlap between the E_C_ of SnO_2_ and the E_V_ of MoO_X_. The quasi-Fermi level of electrons in SnO_2_ aligns closely with that of holes in MoO_X_. In Figure 5d, a current-free region is observed at the SnO_2_/MoO_X_ interface, indicating that charge transport in the recombination layer is dominated by the BBT mechanism. As the carrier concentration in the recombination layer increases from 5 × 10^18^ cm^−3^ to 5 × 10^19^ cm^−3^, the recombination mechanism changes from TAT to BBT. When the charge recombination mechanism is BBT, the tandem solar cell device achieves higher efficiency.

#### 3.2.2. Effect of the Defect Density at the SnO_2_/MoO_X_ Interface

**Figure 6:**
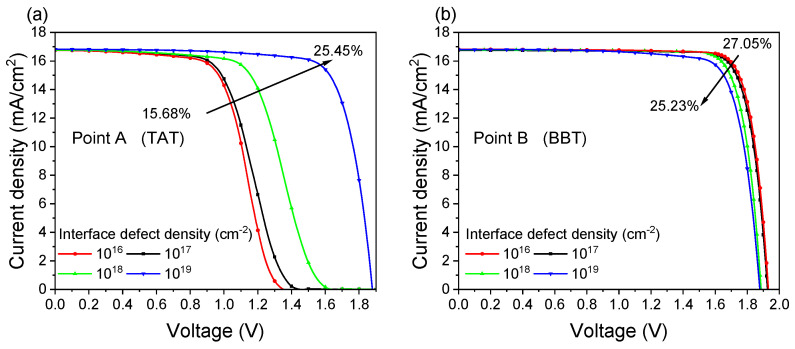
J-V curves under different interface defect densities. (**a**) Point A: SnO_2_/MoO_X_ doping concentration at 5 × 10^18^ cm^−3^. (**b**) Point B: SnO_2_/MoO_X_ doping concentration at 5 × 10^19^ cm^−3^.

This section investigates the impact of the defect density at the SnO_2_/MoO_X_ interface on tandem device performance. Points A and B from the previous section are chosen for the analysis. For point A, as illustrated in Figure 6a, when the defect density at the recombination layer interface increases from 1 × 10^16^ cm^−2^ to 1 × 10^19^ cm^−2^, the cell efficiency is raised from 15.68% to 25.45%. Since the recombination mechanism at point A is dominated by TAT, a high interface defect density significantly enhances electron–hole recombination in the recombination layer, thereby improving the tandem device efficiency. However, for point B, dominated by BBT, increasing the interface defects results in a reduction in tandem cell efficiency. As illustrated in Figure 6b, the efficiency decreases from 27.05% to 25.23% when the defect density is changed from 1 × 10^16^ cm^−2^ to 1 × 10^19^ cm^−2^. Therefore, the optimal doping concentrations for SnO_2_ and MoO_X_ are 5 × 10^19^ cm^−3^, and the ideal defect density at the SnO_2_/MoO_X_ interface is 1 × 10^16^ cm^−2^.

#### 3.2.3. Effect of the Thickness of the Recombination Layer

**Figure 7:**
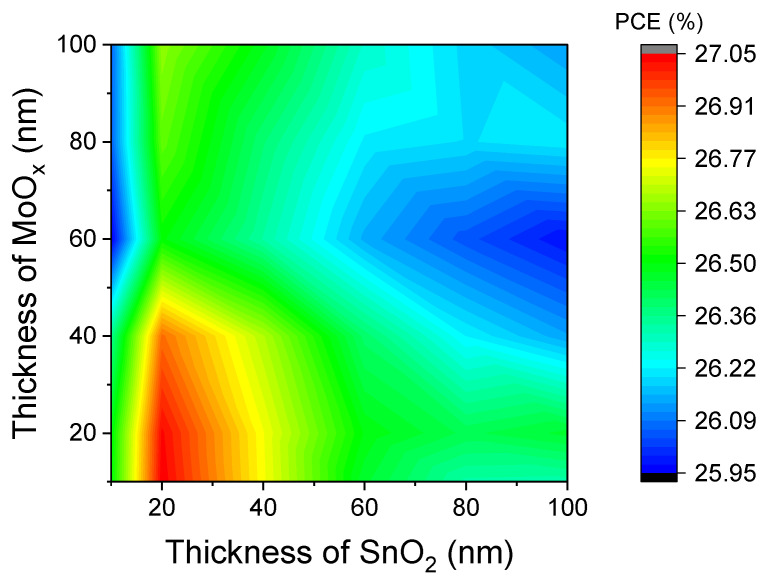
Effect of the thicknesses of SnO_2_/MoO_X_ recombination layer on the PCE of the tandem device.

This section examines the impact of SnO_2_/MoO_X_ recombination layer thickness on tandem cell performance. Figure 7 illustrates the variation in device efficiency as the thicknesses of the SnO_2_ and MoO_X_ layers are adjusted from 10 nm to 100 nm. When the thicknesses of the SnO_2_/MoO_X_ layer are more than 20 nm/20 nm, the PCE declines. Notably, the MoO_X_ layer is dual-functional, and the variations in its thickness can affect both its role as the emitter in the bottom cell and its involvement in electron–hole recombination. The recombination layer exhibits intrinsic optical absorption. Generally, an excessively thick recombination layer reduces cell efficiency due to optical absorption, while an overly thin film not only affects uniform converge but also impairs charge recombination. As shown in Figure 7, the optimal thicknesses for both the SnO_2_ and MoO_X_ layers are approximately 20 nm, consistent with their initial thicknesses. Therefore, the thicknesses of the SnO_2_ and MoO_X_ layers are set to 20 nm each, and the optimized device can achieve a PCE of 27.05%, in agreement with previous results in Section 3.2.2.

### 3.3. Effect of Different HTLs on the Top Cell

**Figure 8:**
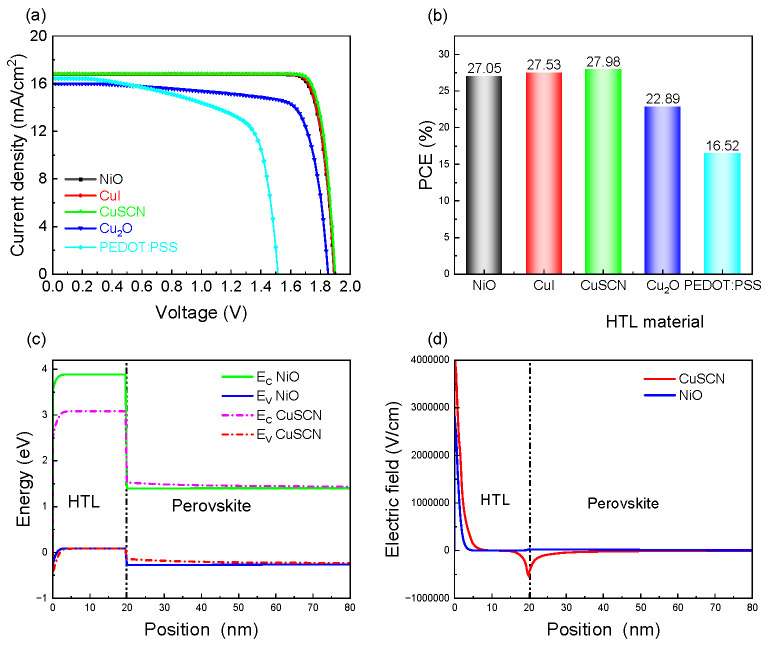
(**a**) J-V curves, (**b**) PCE, (**c**) energy band diagrams, and (**d**) electric field diagrams of tandem cells with NiO and CuSCN.

This section investigates the effect of different HTLs on the top cell on the device performance. The HTLs include NiO, CuI, Cu_2_O, CuSCN, and Poly(3,4-ethylenedioxythiophene)/Poly(styrenesulfonate) (PEDOT:PSS). The relevant parameters for each HTL are shown in Table 3. Figure 8a presents the J-V curves for these HTLs, while Figure 8b shows the corresponding tandem solar cell efficiency, and CuSCN exhibits the best efficiency of 27.98%. To analyze the reasons for the improved cell performance when CuSCN replaces NiO, Figure 8c compares the energy band diagrams of CuSCN and NiO as HTLs. The valence band offset (VBO) represents the difference between the valence band of the HTL and that of the perovskite layer, which is calculated as VBO = E_V_(HTL) − E_V_(perovskite). A negative VBO corresponds to a cliff-like band alignment. As shown in Figure 8c, the NiO/perovskite interface has a cliff of −0.55 eV. The cliff-like band structure does not prevent carrier extraction from the perovskite layer. However, a large cliff significantly increases the recombination rate at the interface [55]. In contrast, CuSCN is used as the HTL, which results in a smaller VBO (−0.45 eV) with the perovskite layer. Thus interface recombination can be reduced, and the efficiency can then be improved from 27.05% to 27.98%. Figure 8d shows the electric field at the HTL/perovskite interface when using CuSCN and NiO as HTLs. With CuSCN as the HTL, the electric field at the interface is broader and stronger, facilitating hole transport. Therefore, CuSCN is chosen as the optimal HTL for the perovskite top cell.

### 3.4. Effect of the Thicknesses of Perovskite and Silicon Absorption Layers

**Figure 9:**
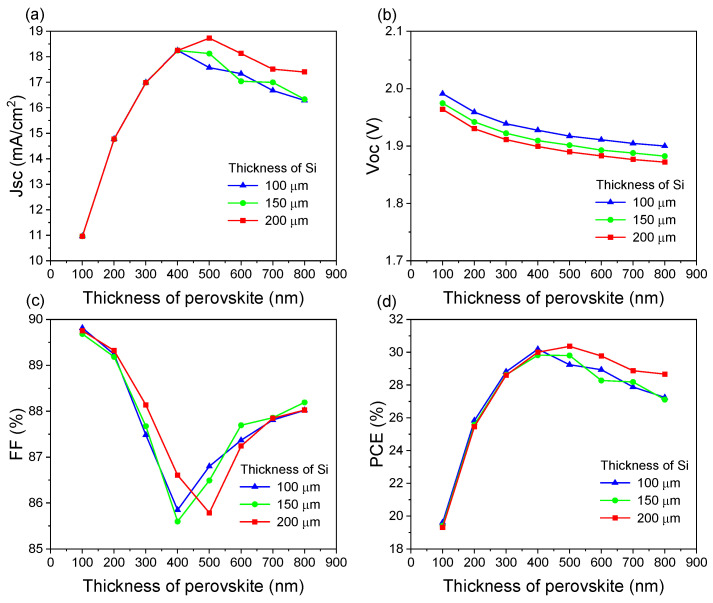
Effect of different top and bottom cell absorber layer thicknesses on tandem cell performance parameters: (**a**) J_SC_, (**b**) V_OC_, (**c**) FF, and (**d**) PCE.

In a tandem device, modifying the absorption layer thickness in the sub-cells causes deviations from the current-matching condition. This deviation significantly affects the photovoltaic characteristics of the tandem device. As shown in Figure 9a, when the perovskite layer thickness increases from 100 nm to 400 nm, the J_SC_ shows an upward trend. However, when the thickness increases from 500 nm to 800 nm, the J_SC_ gradually decreases. When the perovskite layer thickness is below 400 nm, the current is limited by the top cell, and increasing the thickness of the absorption layer in the SHJ bottom cell does not effectively increase the overall current output. On the other hand, when the perovskite layer thickness exceeds 400 nm, the bottom cell becomes the limiting factor for device performance. Therefore, as the SHJ bottom cell absorption layer thickness becomes thicker, the optimal thickness of the top cell absorption layer for maximum J_SC_ also increases. For instance, when the thickness of the bottom cell absorption layer is 100 μm, the optimal perovskite thickness becomes 400 nm, and for the 200 μm silicon absorption layer, it is 500 nm. Correspondingly, the current density increases from 18.1 to 18.7 mA/cm^2^.

Regarding V_OC_, as shown in Figure 9b, increasing the absorption layer thickness slightly decreases V_OC_. As the absorption layer thickness increases, carrier recombination can be enhanced, resulting in V_OC_ reduction. As shown in Figure 9c, FF varies differently from J_SC_. As the perovskite layer thickness increases, FF first decreases, reaching a minimum value at the current-matching point, and then it increases again, suggesting that FF variation is closely related to the degree of current matching between sub-cells [56]. As shown in Figure 9d, the trend in PCE is similar to that of J_SC_. As the perovskite layer thickness increases, PCE first increases and then decreases, primarily due to the increase in J_SC_. When the absorption layer is 500 nm in the top cell and the absorption layer is 200 μm in bottom cell, the tandem device achieves the highest PCE of 30.56% under the condition of current matching.

### 3.5. Effect of the Defect Density of the Perovskite Absorber Layer

**Figure 10:**
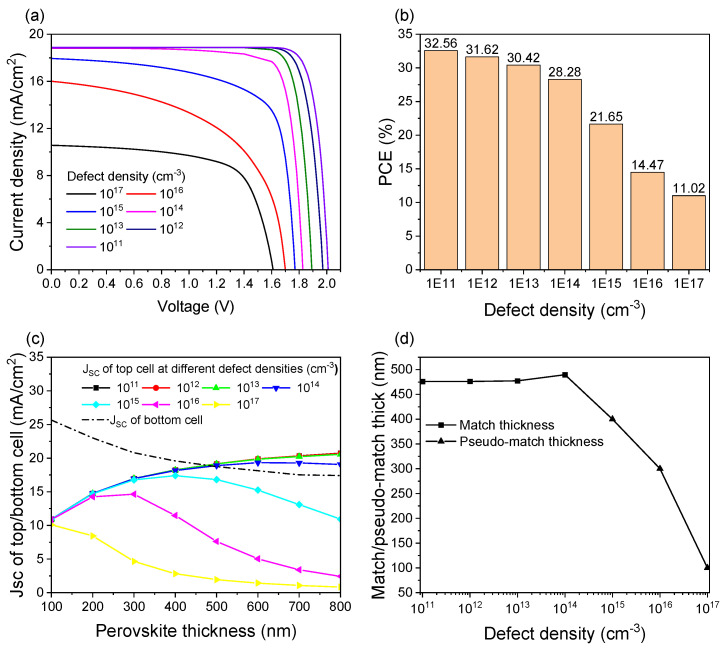
Tandem cell performance under different defect densities (**a**) J-V curves, (**b**) PCE, (**c**) J_SC_ of sub-cells with different perovskite thicknesses, and (**d**) optimal perovskite thickness.

The defect density of the top cell absorber layer has an impact on tandem device performance. Figure 10a demonstrates the J-V curves under varying defect densities of the perovskite absorber layer. Figure 10b shows a significant degradation in tandem cell efficiency from 32.56% to 11.04% as the defect density increases from 10^11^ cm^−3^ to 10^17^ cm^−3^. As shown in Figure 10c, the current-matching point remains achievable through perovskite thickness optimization when the defect density is not more than 10^14^ cm⁻³, as evidenced by the intersection points in their current density curves. However, once the defect density exceeds 10^14^ cm^−3^, the current-matching point is no longer achievable, and the efficiency of the tandem device deteriorates rapidly. Under this non-ideal condition, the J_SC_ of the tandem device is limited by the top cell, and we define the top cell absorber layer thickness at the maximum J_SC_ as the ‘pseudo-matching thickness’ when the J_SC_ difference between sub-cells is the smallest. Figure 10d shows that when the defect density is enlarged from 10^11^ cm^−3^ to 10^14^ cm^−3^, the optimal thickness of the perovskite layer increases from 470 nm to 490 nm under the condition of current matching, and the pseudo-matching thickness decreases after the defect density of 10^14^ cm^−3^. Therefore, when the thickness and the defect density of the perovskite layer are 470 nm and 10^11^ cm^−3^, respectively, the best performance of the tandem cell can reach 32.56%.

### 3.6. Effect of Bottom Cell Texture Structure

**Figure 11:**
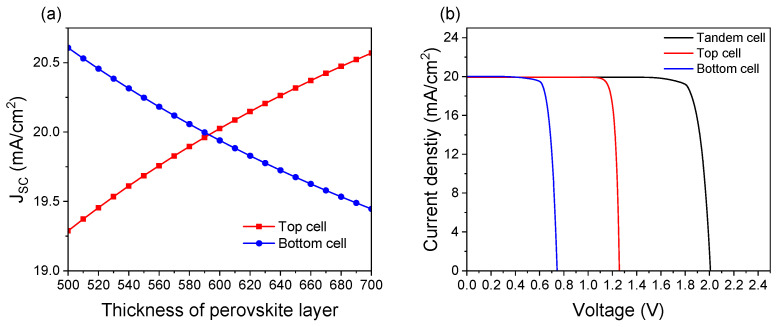
(**a**) J_SC_ of sub-cells as a function of perovskite thickness. (**b**) J-V curves of the tandem and its sub-cells under the condition of current matching.

**Figure 12:**
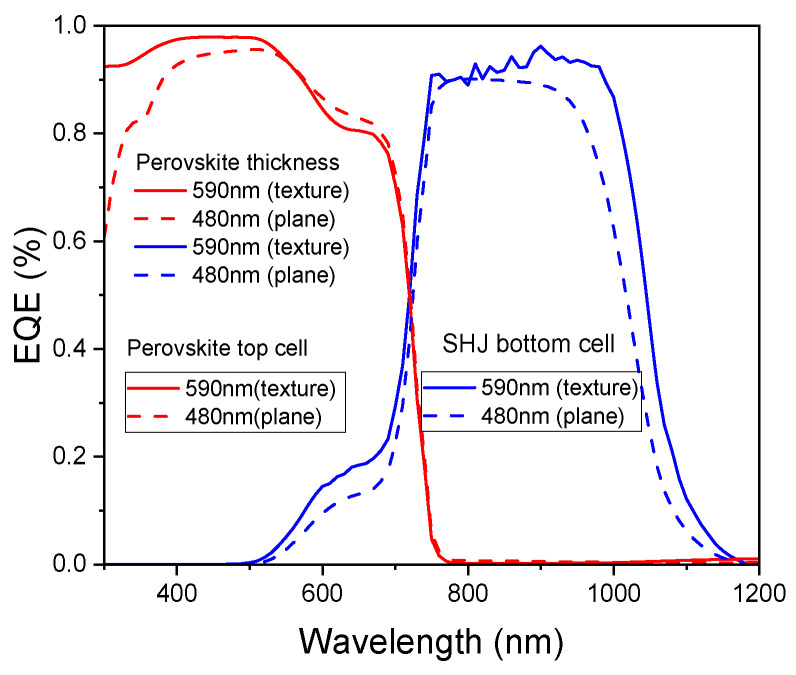
EQE curves of the sub-cells under current-matching point.

Finally, we consider the actual tandem solar cell device structure, in which the perovskite top cell is deposited on the textured SHJ bottom cell. Consequently, the previous planar model is converted into a textured one. The front surface of the SHJ bottom cell features a pyramidal texture, characterized by a pyramid angle of 54.74°, width of 3.536 μm, and height of 5 μm. The crystalline silicon layer in the bottom cell retains a thickness of 200 μm. As shown in Figure 11a, the current-matching point is achieved at a 590 nm perovskite absorber thickness, corresponding to a J_SC_ of 20.23 mA/cm^2^. Figure 11b illustrates the J-V curves of the tandem cell and its sub-cells at the matching point, where the J_SC_ of the sub-cells and the tandem cell are nearly identical. As summarized in Table 5, the tandem cell exhibits a V_OC_ of 2.01 V and a PCE of 35.31%, closely matching the sum of the performance of the two sub-cells. To explore the reason for efficiency improvement by texture structure, Figure 12 compares the external quantum efficiency (EQE) of the textured and planar structures at the current-matching point. The textured structure enhances the absorption in the bottom cell above 900 nm and the top cell below 400 nm, leading to performance improvement.

## 4. Conclusions

This study established a model for perovskite/SHJ tandem solar cells using Silvaco TCAD software. The initial cell structure is ITO/NiO/perovskite/SnO_2_/MoO_X_/i-a-Si:H/n-c-Si/i-a-Si:H/n-a-Si:H/Ag, where MoO_X_ plays dual roles, serving as the emitter for the SHJ bottom cell and participating in charge recombination. First, we investigated the impact of different recombination layers on tandem cell performance, identifying the SnO_2_/MoO_X_ recombination layer as the optimal choice. When the doping concentrations of SnO_2_/MoO_X_ layers are increased from 5 × 10^18^ cm^−3^ to 5 × 10^19^ cm^−3^, the recombination mechanism shifts from the less efficient TAT to the more efficient BBT, leading to significant improvement in tandem solar cell performance. Under the BBT mechanism, the optimal performance is achieved with a defect density of 1 × 10^16^ cm^−2^ at the SnO_2_/MoO_X_ interface and the thicknesses of 20 nm/20 nm, reaching 27.05%. Next, by using CuSCN as the HTL to enhance device performance in the top cell, the efficiency is further improved to 27.82%. Thickness optimization of the top and bottom absorption layers can increase the efficiency to 30.56%. Additionally, by reducing the defect density of the perovskite layer to 10^11^ cm^−3^, the efficiency is raised to 32.56%. Finally, by applying a pyramidal texture to the front surface of the SHJ bottom cell, the tandem cell efficiency reaches 35.31%. These results demonstrate the potential of perovskite/SHJ tandem solar cells with a dual-functional layer of MoO_X_.

## Figures and Tables

**Table 1:** The material parameters of perovskite/SHJ tandem solar cells used in the simulation.

Parameters	NiO	Perovskite	SnO_2_	n-c-Si	i-a-Si:H	n-a-Si:H	MoO_X_
Thickness (um)	0.03	0.3	0.02	100	0.01	0.01	0.02
Bandgap, Eg (eV)	1.4	1.7	9	1.12	1.72	1.72	3
Electron affinity, χ (eV)	3.8	4.05	4.4	4.05	3.86	3.86	2.5
Relative permittivity, ε	10.7	6.5	3.6	11.8	11.8	11.8	5.6
Effective conduction band density, N_C_ (cm^−3^)	2.8 × 10^19^	1 × 10^20^	4 × 10^18^	2.8 × 10^18^	1 × 10^20^	1 × 10^20^	2.8 × 10^18^
Effective valance band density, N_V_ (cm^−3^)	1 × 10^19^	1 × 10^20^	1 × 10^18^	1 × 10^19^	1 × 10^20^	1 × 10^20^	2 × 10^19^
Mobility of electrons, μ_n_ (cm^2^/Vs)	12	8.16	10	1240	20	20	25
Mobility of holes, μ_p_ (cm^2^/Vs)	2.8	8	10	451	5	5	100
Donor doping density, N_D_ (cm^−3^)	-	-	1 × 10^18^	5 × 10^15^	-	1 × 10^18^	-
Acceptor doping density, N_A_ (cm^−3^)	1 × 10^18^	1 × 10^16^	-	-	-	-	1 × 10^18^
Lifetime of electron, τ_n_ (s)	1 × 10^−7^	1 × 10^−6^	1 × 10^−7^	1 × 10^−6^	1 × 10^−5^	1 × 10^−7^	1 × 10^−6^
Lifetime of hole, τ_p_ (s)	1 × 10^−7^	1 × 10^−6^	1 × 10^−7^	1 × 10^−6^	1 × 10^−5^	1 × 10^−7^	1 × 10^−6^

**Table 2:** Defect parameters of the layers and interfaces.

Parameters	Bulk Defects			Interface Defects		
	NiO	Perovskite	SnO_2_	NiO/Perovskite	Perovskite/SnO_2_	SnO_2_/MoO_X_
Defect type	Acceptor	Acceptor	Donor	Acceptor	Acceptor	Neutral
Energy level	Midgap	Midgap	Midgap	Midgap	Midgap	Midgap
Defect density	1 × 10^16^ cm^−3^	1 × 10^14^ cm^−3^	1 × 10^16^ cm^−3^	1 × 10^13^ cm^−2^	1 × 10^13^ cm^−2^	1 × 10^18^ cm^−2^

**Table 3:** The material parameters of HTLs.

Parameters	CuI	CuSCN	Cu_2_O	PEDOT:PSS
Thickness (nm)	20	20	20	20
Bandgap, Eg (eV)	2.1	3.6	2.1	2.2
Electron affinity, χ (eV)	2.98	1.7	3.2	2.9
Relative permittivity, ε	6.5	10	7.1	3
Mobility of electrons, μ_n_ (cm^2^/Vs)	100	100	111	0.02
Mobility of holes, μ_p_ (cm^2^/Vs)	43.9	25	80	0.02
Acceptor doping density, N_A_ (cm^−3^)	1 × 10^18^	1 × 10^18^	1 × 10^18^	1 × 10^18^
Effective conduction band density, N_C_ (cm^−3^)	2.8 × 10^19^	2.2 × 10^19^	2.5 × 10^19^	2.2 × 10^15^
Effective valance band density, N_V_ (cm^−3^)	1 × 10^19^	1.8 × 10^18^	1.8 × 10^19^	2.2 × 10^19^

**Table 4:** Tandem cell performance with different recombination layers.

Recombination Layer	J_SC_ (mA/cm^2^)	V_OC_ (V)	FF (%)	PCE (%)
ITO	15.45	1.81	88.04	24.63
p^+^-nc-Si:H/n^+^-nc-Si:H	16.51	1.89	79.31	24.78
SnO_2_/MoO_X_	16.82	1.87	80.5	25.45
SnO_2_/ITO/MoO_X_	15.72	1.81	87.9	25.04

**Table 5:** Performance parameters of top, bottom, and tandem cell.

	J_SC_ (mA/cm^2^)	V_OC_ (V)	FF (%)	PCE (%)
Perovskite top cell	20.23	1.266	88.84	22.75
SHJ bottom cell	20.30	0.742	85.05	12.81
Perovskite/SHJ tandem cell	20.23	2.008	86.92	35.31

## Data Availability

The original contributions presented in this study are included in the article. Further inquiries can be directed to the corresponding author.
